# The Mole Mapper Study, mobile phone skin imaging and melanoma risk data collected using ResearchKit

**DOI:** 10.1038/sdata.2017.5

**Published:** 2017-02-14

**Authors:** Dan E. Webster, Christine Suver, Megan Doerr, Erin Mounts, Lisa Domenico, Tracy Petrie, Sancy A. Leachman, Andrew D. Trister, Brian M. Bot

**Affiliations:** 1 National Cancer Institute, Bethesda, Maryland 20892, USA; 2 Sage Bionetworks, Seattle, Washington 98109, USA; 3 Oregon Health & Science University, Portland, Oregon 97239, USA

**Keywords:** Melanoma, Risk factors, Research data

## Abstract

Sensor-embedded phones are an emerging facilitator for participant-driven research studies. Skin cancer research is particularly amenable to this approach, as phone cameras enable self-examination and documentation of mole abnormalities that may signal a progression towards melanoma. Aggregation and open sharing of this participant-collected data can be foundational for research and the development of early cancer detection tools. Here we describe data from Mole Mapper, an iPhone-based observational study built using the Apple ResearchKit framework. The Mole Mapper app was designed to collect participant-provided images and measurements of moles, together with demographic and behavioral information relating to melanoma risk. The study cohort includes 2,069 participants who contributed 1,920 demographic surveys, 3,274 mole measurements, and 2,422 curated mole images. Survey data recapitulates associations between melanoma and known demographic risks, with red hair as the most significant factor in this cohort. Participant-provided mole measurements indicate an average mole size of 3.95 mm. These data have been made available to engage researchers in a collaborative, multidisciplinary effort to better understand and prevent melanoma.

## Background & Summary

Melanoma is a cancer that arises from pigment-producing melanocytes in the skin. Clusters of melanocytes known as melanocytic nevi, or moles, can be present at birth or can emerge throughout childhood and young adulthood and are considered to be benign neoplasms. However, inherited or acquired DNA mutations–in particular, those caused by ultraviolet radiation exposure^1^–can drive malignant transformation to melanoma. The risk of developing melanoma varies widely depending on genetic, demographic, and behavioral factors^2^. A family history of melanoma, fair skin and hair, a large number of moles, and even profession^3^ can contribute to an increased overall risk.

Melanoma is the deadliest cancer of the skin, but early detection results in a highly significant difference in survival: the 10-year survival rate for the earliest stage melanoma (stage IA) is 93%, whereas for the latest stage it is only 10–15% (ref. 4). The significant survival benefit conferred by early detection is the rationale for behavioral interventions aimed at increasing skin self-examination^5^ to recognize changes to existing moles or identify newly emergent moles. Mobile phones with high-resolution cameras can aid these efforts, providing a convenient tool for self-directed skin examination and documentation of the results. In addition, recent innovations in mobile technology allow for self-tracking data to be remotely contributed by individuals and aggregated for biomedical research studies that investigate normal variation and disease symptoms across a large population^6^.

In October 2015, we launched the Mole Mapper Study, an iPhone-based observational study with an accompanying app built using Apple’s ResearchKit framework (http://researchkit.org/). The Mole Mapper iOS app enables participants to contribute measurements and images of their moles as well as answer demographic and behavioral questions related to melanoma risk. The study app was specifically designed not to provide medical advice. During the consent process, participants were required to acknowledge this by answering a quiz question to demonstrate they understand that Mole Mapper ‘doesn’t provide medical care... and doesn’t provide diagnosis or treatment information’. As Mole Mapper is explicitly not meant to recommend or replace in-clinic examination, our study doesn’t include re-contact of users whose mole image suggests a possible malignant skin lesion.

**Figure 1:**
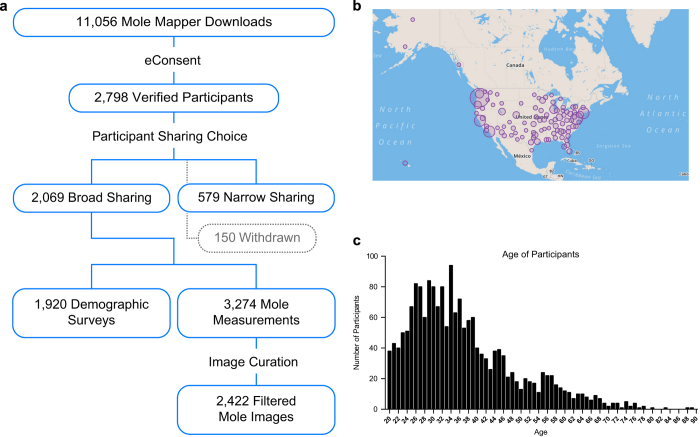
Mole Mapper Melanoma Study cohort description. (**a**) Number of users at each stage of the study registration and data sharing workflow and the principal data types shared. (**b**) Geographic distribution of registered users in the US. (**c**) Age distribution of registered participants.

Full app functionality was provided to all users independent of study enrollment status. From October 15, 2015 to June 1, 2016, the Mole Mapper iOS app was downloaded by 11,056 users, of which 2,798 (25.3%) chose to enroll and contribute their data to the study (Fig. 1a). Study participants self-selected their data sharing preference, either with qualified researchers worldwide (‘broad sharing’) or only with the Mole Mapper study team and its partners (‘narrow sharing’). The study cohort described here is composed of contributions from study participants who designated broad sharing of their data (*n*=2,069).

**Figure 2:**
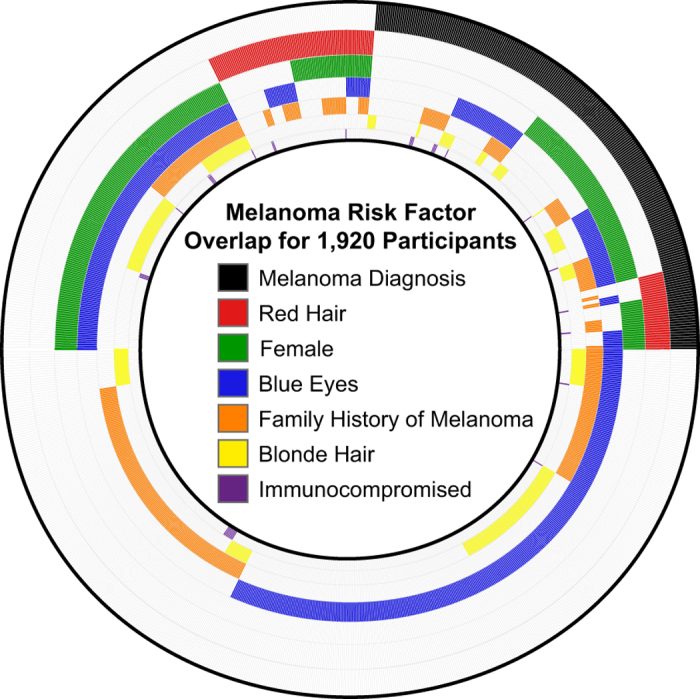
Demographic melanoma risk factors in the cohort. Venn Piagram representation of melanoma risk factors in the cohort and their overlap.

The study cohort includes data from participants across the US (Fig. 1b) that span a wide age range (Fig. 1c). Demographic survey data documents several known melanoma risk factors within the cohort (Fig. 2, Table 1). We observed that self-reported data recapitulates the expected association^7^ between individuals with red hair color and melanoma diagnosis (Table 1), supporting the of the mobile-based self-reporting survey instruments.

**Figure 3:**
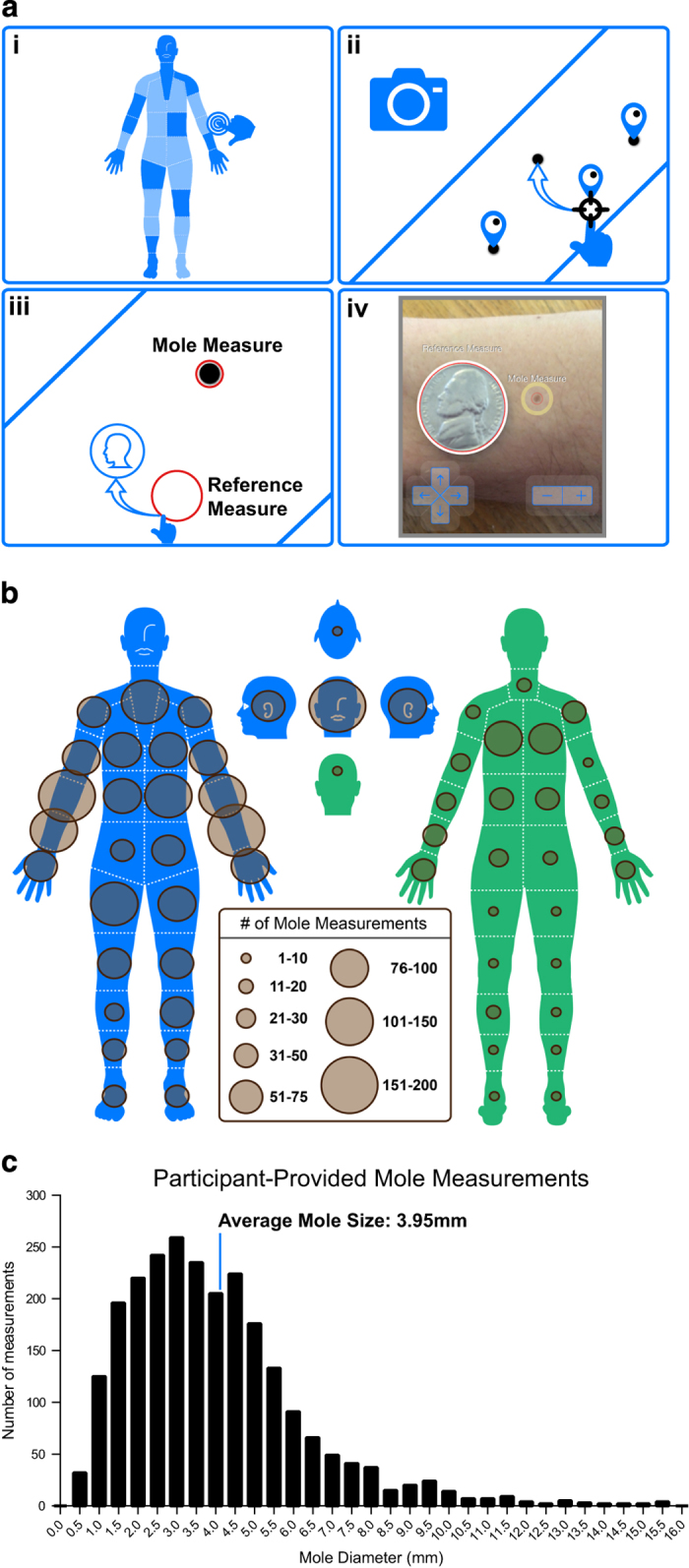
Participant-provided mole measurements. (**a**) Schematic representation of mole measurement workflow in the Mole Mapper app. (**b**) Distribution of mole measurements in the cohort in each of the body map zones (**c**) Distribution of participant-provided mole measurements.

The Mole Mapper app quantitatively measures mole size by comparison to a reference item of known size, such as a coin, to enable an absolute measurement in millimeters (Fig. 3a). We defined 59 body zones in which to map mole location. Across this cohort, all 59 zones were documented, with the greatest number of moles measured on the forearms and face (Fig. 3b). The curated participant-provided measurements indicate an average mole size of 3.95 mm (median 3.56 mm, IQR 2.33–5.01) (Fig. 3c). Repeated measurements were taken for 291 moles of the 2,868 total moles (10.1%) measured in the cohort. These repeated measurements and images represent an opportunity to assess variance in mole size change over time, and we anticipate that this resource will become more valuable as study participants continue to add longitudinal data.

The Mole Mapper Study is ongoing, with continued development and iterative improvements based on this initial stage of this project. Future directions of the study include implementation of automated mole detection, integration of genetic testing results, inclusion of the Android operating system, and extension outside of the United States.

The Mole Mapper Study takes a participant-centered approach, providing participants with control over data generation and data sharing. This public data release and the open source nature of the app code is in keeping with the commitment we have made to participants to maximize the utility of the data they generously contribute. We hope to engage researchers across many disciplines, including computer vision, dermatology, machine learning, cancer biology, and epidemiology, to better understand normal skin health and ultimately reduce melanoma-related mortality.

## Methods

### Participant Onboarding

The Mole Mapper Study app was made available in the United States beginning in October 2015 through the Apple App Store. An iPhone 4S or newer running iOS 8 or newer was required to download the app. Enrollment was open to all individuals over age 18 with a US registered iPhone. Prospective participants proceeded through an interactive eConsent process (http://sagebase.org/pcc/) utilizing the ResearchKit framework (http://researchkit.org/). Participants were required to correctly answer a 5-question quiz relating to their understanding of potential risks of contributing photos, the study aims, data security, participant rights, and data sharing options. Participants electronically signed an informed consent form. Participants then registered with the Sage Bionetworks Bridge Server infrastructure using an email address and password. This email address was used for study enrollment verification and receipt of the signed consent form. Ethical oversight of the study was obtained from Western Institutional Review Board (Protocol #20151976).

Participants were given the option to either share their data only with the Mole Mapper Study Team and partners (‘share narrowly’) or to share their data more broadly with qualified researchers worldwide. The data presented here consist of all individuals who chose to have their data shared broadly.

### Demographic and Monthly Surveys

Directly after study registration, participants are asked to complete a one-time survey to provide demographic and medical information about themselves relating to factors that may contribute to melanoma risk, including self reported melanoma diagnosis and melanoma diagnosis for any blood relatives (Table 2). Once each month during the study, participants are prompted upon opening the app to answer 5 questions about the previous month relating to their behavior or medical status over the previous month (Table 3). All questions in the surveys are optional for the participant to complete.

### Participant-Provided Mole Measurements

The workflow for measuring a mole (Fig. 3a) begins by selecting a location on the body within any of 59 distinct body map zones (Supplementary Table 1). These zones represent a portion of the body that can be captured by a typical mobile phone camera held at less than an arm’s length away.

Participants are instructed to take a photo of this entire zone, which may contain multiple moles. After photos are taken, moles within the specified zone are identified by the user by dropping a pin and dragging it over the mole. Participants select the mole they want to measure by tapping on a pin and subsequently taking a photo of this mole adjacent to a reference item of known length. US coins are presented as a default set of reference items, however participants can provide any reference of known length, such as a USB connector or ruler.

The central 320×568 pixel frame of the photo is presented to the participant overlain with 2 circular measurement tools: one for the mole and one for the reference item. Participants are instructed to drag and resize the circles such that they circumscribe the mole and reference item, respectively. The diameter of the circles in pixel size, together with the known value of the reference item in millimeters are used to calculate an absolute mole diameter in millimeters using the following formula:

Mole Diameter in mm=(Reference Item Diameter in mm×Mole Diameter in pixels)/Reference Item Diameter in pixels

### Image Curation and Censoring

Images were assessed by at least two independent curators using the criteria outlined below for identifiable or invalid images. If either of the 2 curators determined an image as identifiable or invalid, it was flagged as such in the dataset. If the image was deemed identifiable, measurement information as calculated by the app is maintained, but the image is excluded from the data release.

The following criteria were used for determining an identifiable image (852/3274, 26% of total images submitted):

1. An image that contains a person’s eyes
2. An image that contains ~50% of the face, including mouth and nose, or a full ear
3. An image that has distinctive jewelry, tattoos, or 2 or more unique background patterns
4. An image that has background items that would be identifiable such as another person, handwriting, or signage that may indicate a location

The following criteria were used to determine an invalid image (321/3274, 10% of total images submitted):

1. An image that is too blurry to make out a mole or reference item
2. An image that does not contain skin, such as a practice picture of the room
3. An image that does not contain a reference item to determine absolute size

### Data Collection and Distribution

The app records all data on-device within the sandboxed storage location for the app. If the participant consented to the study, data is encrypted and sent to the Bridge Server, a set of web services developed and operated by Sage Bionetworks (http://sagebase.org/)^7^. Bridge exposes RESTful API to collect and manage mobile health data. This includes secure account creation, consent tracking, and collection of other personal information separately from coded study data intended to be shared with research teams. There are two parts to the encrypted data that is transferred from the participant’s phone to the server: identifiable information upon enrolment (email, first/last name, consent record) and the non-identifiable study data (mole measurements, demographics, etc.). These two components are stored separately and linked by an encrypted account identifier and a healthCode, which indicates a study participant in the coded data (https://developer.sagebridge.org/articles/security.html).

Coded study data, consisting of survey responses and image data, is exported to Synapse (https://www.synapse.org/) for distribution to researchers. Synapse^8^ is a general-purpose data and analysis sharing platform where users can work collaboratively, analyze data, share insights and have attributions and provenance of those insights to share with others.

### Code Availability

The Mole Mapper iOS app (https://github.com/Sage-Bionetworks/MoleMapper) was built using Apple’s ResearchKit framework (http://researchkit.org/), which is open source and available on GitHub (https://github.com/researchkit/researchkit). The Bridge iOS SDK (https://github.com/Sage-Bionetworks/Bridge-iOS-SDK) provides integration with Sage Bionetworks’ Bridge Server, a back-end data service designed for collection of participant donated study data (https://developer.sagebridge.org/). The Mole Mapper app can be downloaded on the Apple App Store at (https://itunes.apple.com/us/app/mole-mapper-melanoma-study/id1048337814?mt=8).

## Data Records

Upon enrollment in the study, participants were presented with a one-time demographic survey with questions relating to factors that can influence the risk for developing melanoma (Data Citation 1; Table 2). Each month after enrollment, a monthly follow-up survey (Data Citation 2; Table 3) was presented that asked users about their activity and health over that past month. All questions were optional and could be skipped with no information entered.

Participants were able to map the location and measure the size of moles on their skin using a workflow described in the Participant-Provided Mole Measurements section. The mole measurement tools were presented on screen as perfect circles that can be re-sized to circumscribe the mole and the reference item. Each mole is auto-assigned a mutable name that is presented in the user interface, and an immutable integer value that is used for data aggregation. Mole ID integer values are redundant within the entire cohort dataset. To achieve a unique identifier for a given mole, the participant healthCode must be combined with the mole ID. Participant-provided mole measurements (Data Citation 3) denote the diameter of the calculated mole size in millimeters.

Participant-provided measurements contained over-represented and potentially artifactual data resulting from default diameters that have not been adjusted by the participant. The default diameter values are denoted in the dataset (Data Citation 3) for the following reference items: US penny, US dime, US quarter.

Images from the mole measurements (Data Citation 4) have been curated following the procedure detailed in the Image Curation and Censoring section. If an image was deemed to be possibly identifiable, all metadata pertaining to that measurement remains in the dataset, but the image itself was removed from the dataset. An image is flagged as invalid if it is not a photo of a mole or does not contain a reference item by which to quantify the mole size.

If a user indicated that they had a mole removed, they were asked to denote which mole was removed, and to take a photo of the biopsy site after healing (Data Citation 5). Photos of these moles are stored together with all mole images (Data Citation 3).

## Technical Validation

To determine the possible accuracy of measurements made with the Mole Mapper app, 4 black circles were created on a piece of paper with the following diameters: 1 mm, 2 mm, 4 mm, 8 mm. These circles were measured with 4 different reference items (a US penny, US nickel, US dime, US quarter) and the app-provided, calculated diameter was plotted against the known standards. The R^2^ value for this correlation was 0.9983, with a deviation from the known standard at any of the given references of less than 0.1 mm. (Supplementary Fig. 1, Supplementary Table 2). It is important to note that the measurements described above were obtained by a user with complete understanding of the provided measurement instructions. Deviations from the intended workflow (i.e. switching reference item and measurement circles) will provide inaccurate measurements.

## Usage Notes

We have instituted governance structures that balance sharing these data for secondary research with commensurate privacy protections for participants.

Researchers interested in accessing these data will need to complete the following steps:

1. Register for a Synapse account (www.synapse.org)
2. Become a Synapse Certified User by passing a short quiz (www.synapse.org/#!Quiz:Certification)
3. Have their Synapse User Profile validated by the Synapse Access and Compliance Team (ACT)
4. Submit an Intended Data Use statement that is publically posted
5. Agree to the data-specific Conditions for Use (see DOIs for each data source)

While certain data types may have additional Conditions for Use (e.g. mole images), the overarching Conditions for Use are as follows:

- You confirm that you will not attempt to re-identify research participants for any reason, including for re-identification theory research
- You reaffirm your commitment to the Synapse Awareness and Ethics Pledge
- You agree to abide by the guiding principles for responsible research use and data handling as described in the Synapse Governance documents
- You commit to keeping these data confidential and secure
- You agree to use these data exclusively as described in your submitted Intended Data Use statement
- You understand that these data may not be used for commercial advertisement or to re-contact research participants
- You agree to report any misuse or data release, intentional or inadvertent, to the ACT within 5 business days by emailing act@sagebase.org
- You agree to publish findings in open access publications
- You promise to acknowledge the research participants as data contributors and study investigators on all publication or presentation resulting from using these data as follows: ‘These data were contributed by users of the MoleMapper mobile application as part of the MoleMapper study developed by Sage Bionetworks and OHSU, and described in Synapse [doi:10.7303/syn5576734].’

Due to the potentially sensitive nature of images provided as part of the Mole Mapper study, in addition to the steps outline above, we require that researchers wishing to work with these images have their research plan reviewed by an accredited ethics board/IRB. Full information about how to access the data is available in Synapse (www.synapse.org/molemapper).

## Additional Information

**How to cite this article**: Webster, D. E. *et al.* The Mole Mapper Study, mobile phone skin imaging and melanoma risk data collected using ResearchKit. *Sci. Data* 4:170005 doi: 10.1038/sdata.2017.5 (2017).

**Publisher’s note**: Springer Nature remains neutral with regard to jurisdictional claims in published maps and institutional affiliations.

## Supplementary Material



Supplementary Figure 1

Supplementary Table 1

Supplementary Table 2

## Figures and Tables

**Table 1:** Self-identified demographic risk factors and association with reported melanoma diagnosis.

**Melanoma risk factor**	**Overall (*****n*****=1,920)**	**No melanoma diagnosis (*****n*****=1,688)**	**Melanoma diagnosis (*****n*****=232)**	**Pearson’s Chi-Squared** ***P*****-value**
*Hair Color, %*				
Red	6.3	5.2	13.9	<0.0001
Blonde	20.8	20.4	23.8	
Brown	62.5	63	58.9	
Black	10.5	11.4	3.5	

*Gender, %*				
Female	41.7	40	53.7	0.0003
Male	55.9	57.5	43.7	
Other	2.5	2.5	2.6	

*Eye Color, %*				
Blue	35.3	34	44.9	0.001
Green	23.2	23	24	
Brown	41.6	43	31.1	

Family History of Melanoma, %	28.3	27.5	33.6	0.06
Immunocompromised, %	3.2	3	4.7	0.23
Autoimmune Disease, %	9.1	8.9	10.3	0.55

**Table 2:** Description of baseline demographic survey data.

**Question**	**Variable name**	**Variable details**
What is your date of birth?	birthYear	Integer (year of birth)
What is your gender?	gender	One of {Female, Male, Other}
What is your zip code?	shortenedZip	Integer (truncated to first 3 digits)
What is your natural hair color?	hairColor	One of {Black hair, Brown hair, Blonde hair, Red hair}
What is your natural eye color?	eyeColor	One of {Brown eyes, Green eyes, Blue eyes}
Have you worked in any of the following professions?	profession	One of {Pilot or flight crew,Dental professional, Construction, Radiology technician, Farming, TSA Agent, Coal/Oil/Gas extraction, Military veteran, Doctor/Nurse, Welding/Soldering, Electrician, Biomedical researcher, none of the above}
Have you ever been diagnosed with Melanoma?	melanomaDiagnosis	One of {True, False}
Has a blood relative (parent, sibling, child) ever had Melanoma?	familyHistory	One of {True, False}
Have you ever had a mole removed?	moleRemoved	One of {True, False}
Do you have an autoimmune condition (Psoriasis, Chron's Disease, or others)?	autoImmune	One of {True, False}
Do you have a weakened immune system for any reason (transplant recipient, Lupus, prescribed drugs that suppress the immune system)?	immunocompromised	One of {True, False}

**Table 3:** Description of follow-up survey data.

**Question**	**Variable name**	**Variable details**
In the last month, have you gotten a tan?	tan	One of {True, False}
In the last month, have you been sunburned?	sunburn	One of {True, False}
In the last month, have you used sunscreen?	sunscreen	One of {True, False}
In the last month, have you become sick or ill enough to miss work or school?	sick	One of {True, False}
In the last month, have you had any moles removed?	moleRemoved	One of {True, False}
