# Supplementary material for: The Mole Mapper Study, mobile phone skin imaging and melanoma risk data collected using ResearchKit
Source: Sci Data. 2017 Feb 14;4:170005. doi: 10.1038/sdata.2017.5 (PMC5308198; doi:10.1038/sdata.2017.5)
Supplement: Supplementary Figure 1 [file sdata20175-s2.pdf]

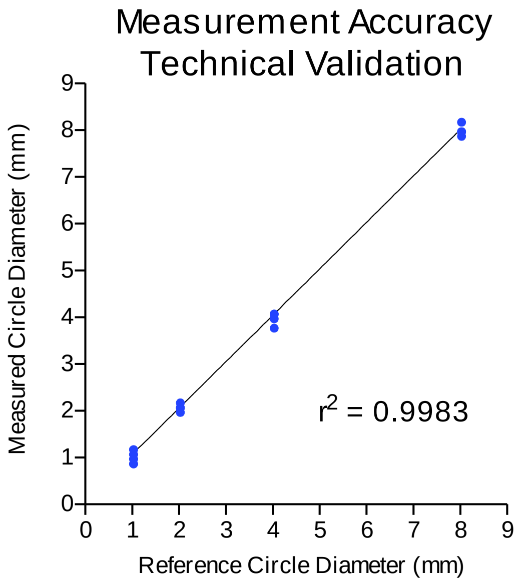

Supplementary Figure 1: Technical validation of mole measurement accuracy by plotting app-derived measurements of circles against the known diameters. Replicate measurements were made using multiple US coins as reference items
